# Supplementary material for: Influence of Tree Species and Size on Bark Browsing by Large Wild Herbivores
Source: Plants (Basel). 2022 Oct 30;11(21):2925. doi: 10.3390/plants11212925 (PMC9653979; doi:10.3390/plants11212925)
Supplement: Supplementary file 1 [file plants-11-02925-s001.zip › plants-1991533-supplementary.pdf]

plants

MDPI

Supplementary material

Table S1. Model of bark thickness (see also [33]), specifically expressing relationship between bark thickness T_b and diameter D_0 as well as distance from the ground (Hg) for three broadleaved tree species specifically common aspen (*Populus tremula* L.), common rowan (*Sorbus aucuparia* L.) and goat willow (*Salix caprea* L.), described by the formula: $T_b = b_0 d_0^{b1} H_g^{b2}$ (where T_b is in mm, D_0 in mm, H_g in cm) where b_0 , b_1 , b_2 are parameters, their standard errors (S.E.), p-value (P), coefficient of determination (R^2), mean squared error (MSE). The models were made from sampling trees collected in a variety of sites in Slovakia.

Tree species	b_0	S.E.	P	b_1	S.E.	P	b_2	S.E.
Common aspen	0.092	0.016	<0.001	0.968	0.016	<0.001	-0.144	0.004
Common rowan	0.160	0.011	<0.001	0.620	0.018	<0.001	-0.055	0.005
Goat willow	0.129	0.010	<0.001	0.784	0.023	<0.001	-0.145	0.004
